# Supplementary material for: Mapping the evolution of fertility support policies in China: A content and instrumental analysis
Source: PLoS One. 2025 Oct 9;20(10):e0332137. doi: 10.1371/journal.pone.0332137 (PMC12510515; doi:10.1371/journal.pone.0332137)
Supplement: S1 Appendix — (ZIP) [file pone.0332137.s001.zip › S1 Appendix. 226 original policy documents/177-中共中央、国务院关于加强人口与计划生育工作稳定低生育水平的决定(FBM-CLI-16-27163).doc]

中共中央、国务院关于加强人口与计划生育工作稳定低生育水平的决定

**发布部门：** [中国共产党中央委员会](https://www.pkulaw.com/law/chl?Aggs.IssueDepartment=a01) [国务院](https://www.pkulaw.com/law/chl?Aggs.IssueDepartment=5)

**发文字号：**中发〔2000〕8号

**发布日期：**2000.03.02

**实施日期：**2000.03.02

**时效性：** [现行有效](https://www.pkulaw.com/law/chl?Aggs.TimelinessDic=01)

**效力级别：** [党内法规制度](https://www.pkulaw.com/law/chl?Aggs.EffectivenessDic=XR12)

**法规类别：** [人口计划管理](https://www.pkulaw.com/law/chl?Aggs.Category=09701)

　　　　　　　　　　　中共中央　国务院关于加强人口
　　　　　　　　　 与计划生育工作稳定低生育水平的决定
　　　　　　　（2000年3月2日　中发〔2000〕8号）

　　人口问题是社会主义初级阶段长期面临的重大问题，是制约我国经济和社会发展的关键因素。控制人口数量，提高人口素质，是实现我国社会主义现代化建设宏伟目标和可持续发展的重大战略决策。新中国成立50年来，特别是改革开放以来，经过全党全国人民的艰苦努力，我国人口与计划生育工作取得举世瞩目的成就。在经济还不发达的情况下，有效地控制了人口过快增长，使生育水平下降到更替水平以下，实现了人口再生产类型从高出生、低死亡、高增长到低出生、低死亡、低增长的历史性转变，成功地探索了一条具有中国特色的综合治理人口问题的道路，有力地促进了综合国力的提高、社会的进步和人民生活的改善，对稳定世界人口做出了积极贡献。
　　计划生育是我们必须长期坚持的基本国策。在实现了人口再生产类型的转变之后，人口与计划生育工作的主要任务将转向稳定低生育水平，提高出生人口素质。全党全社会必须从我国社会主义现代化建设的大局和中华民族生存与发展的长远利益出发，进一步抓紧抓好人口与计划生育工作。为此，现作出如下决定。
　　**一、**稳定低生育水平是今后一个时期重大而艰巨的任务
　　1．随着21世纪的到来，我国人口与计划生育事业将进入一个新的重要发展时期。未来几十年，在实现稳定低生育水平的前提下，我国人口将由低增长逐步过渡到零增长，人口总量达到峰值后（接近16亿）开始缓慢下降，人口素质不断提高，为基本实现现代化和可持续发展创造良好的人口环境。
　　2．人口过多仍是我国首要的问题。未来十几年，我国人口数量还将持续增长，预计年均净增1000万人以上，人口素质不高的状况短期内难以根本改变，劳动就业压力进一步加大，人口老龄化问题更加突出，人口与经济、社会、资源、环境之间的矛盾依然尖锐。
　　稳定低生育水平的工作要求更高，任务更艰巨。目前，我国生产力水平较低，地区发展不平衡，社会保障制度尚不健全，传统生育观念的影响还将长期存在，实行计划生育仍有相当难度。任何政策的偏差、工作的失误以及外部环境的不利影响，都可能导致生育率的回升。在社会主义市场经济体制逐步建立的过程中，人口与计划生育工作面临许多新情况、新问题，管理体制、工作方法、服务质量以及干部素质不能完全适应形势的发展，迫切需要转变政府职能，加快改革和创新的步伐。
　　3．未来十年是稳定低生育水平的关键时期。应当指出，目前还有相当一部分同志对人口多是我国社会主义初级阶段最基本、最重要的国情认识不足，对可持续发展战略中人口问题是关键认识不足，对社会主义市场经济条件下人口与计划生育工作的长期性、艰巨性、复杂性认识不足。切实转变观念，解决这些认识问题，克服盲目乐观、麻痹松懈情绪，是当务之急。
　　4．人口问题的本质是发展问题。能否解决好人口问题，直接关系到人民生活改善、全民素质提高和中华民族的兴衰。各级领导干部必须从全局和战略的高度认识和解决人口问题，以对党和国家、对群众利益、对子孙后代高度负责的精神，保持清醒头脑，坚持科学的态度，一年接一年，坚持不懈地把人口与计划生育工作抓紧抓好，确保未来人口与计划生育目标的实现。

　　**二、**今后十年人口与计划生育工作的目标和方针
　　5．今后十年人口与计划生育工作的目标是：到2010年末，全国人口总数（不含香港、澳门特别行政区和台湾省）控制在14亿以内，年均人口出生率不超过15‰；出生人口素质明显提高；出生婴儿性别比超向正常；育龄群众享有基本的生殖保健服务，普遍开展避孕节育措施的“知情选择”；初步形成新的婚育观念和生育文化；逐步建立调控有力、管理有效、政策法规完备的计划生育保障体系和工作机制。
　　6．为实现上述目标，必须坚持以下方针：
　　－－人口与发展综合决策。加快经济和社会发展，高度重视科技和教育，努力提高人民生活水平和国民素质，把人口与计划生育工作纳入经济和社会发展的总体规划，制定完善各项配套决策，促进人口与经济、社会、资源、环境协调发展。
　　－－稳定现行的生育政策。国家鼓励晚婚晚育，提倡一对夫妻生育一个子女，依照法律法规合理安排生育第二个子女。少数民族也要实行计划生育。具体政策规定由各省、自治区、直辖市制定。
　　－－综合治理人口问题。动员全社会力量，建立政府领导、部门指导、各方配合、群众参与的工作机制，做到优势互补、资源共享、各负其责。采取法律、教育、经济、行政等措施综合治理人口问题。
　　－－国家指导与群众自愿相结合。国家制定政策并提供必要的保障措施，兼顾国家利益和个利益、长远利益和近期利益、整体利益和局部利益，实行行政管理与群众工作相结合，以促进群众生育观念的根本转变为立足点，组织和引导群众积极参与人口与计划生育工作，提高群众实行计划生育的自觉性，进一步密切党群、干群关系。
　　－－整体推进与分类指导相结合。在抓好全国计划生育工作的同时，继续把工作重点放在农村特别是中西部地区的农村。发挥城市和东部地区的示范作用。进一步落实宣传教育为主、避孕为主、经常性工作为主的“三为主”方针，推动不同地区人口与计划生育工作均衡发展。
　　－－以人的全面发展为中心。尊重人民群众作为计划生育主人的地位，维护其合法权益。把计划生育工作与发展经济、帮助群众勤劳致富、建设文明幸福家庭有机结合起来。依靠科技进步，提供优质服务。

　　**三、**完善人口与计划生育工作的调控体系和相关社会经济政策
　　7．建立完备的调控体系和良好的政策环境，是做好人口与计划生育工作的重要保障。各级政府和有关部门制定土地、企业、医疗、社会保障、户籍、劳动、教育、财税等制度和改革措施，要统筹考虑，相互协调，有利于人口与计划生育工作。
　　改进和完善计划生育管理，充分发挥人口计划的调控作用。计划部门要做好人口与经济社会协调发展的规划。公安部门要切实加强出生登记，认真做好公民身份号码编制工作。计划生育部门要制定科学规范的工作制度。乡、村两级按现行政策进行管理，简化手续，公开办事程序和政策，自觉接受群众监督。
　　8．加强人口与计划生育工作的法制建设，加快人口与计划生育国家立法进程，逐步建立健全人口与计划生育法制体系。加强计划生育法制宣传，增强干部群众的法律意识。提高依法行政水平，严格执法，文明执法，充分发挥群众监督和舆论监督的作用，切实维护群众的合法权益，使人口与计划生育工作全面走上依法管理的轨道。
　　9．建立和完善计划生育利益导向机制。各级政府及涉农等部门要采取小额贷款、项目优先、科技扶持、政策优惠等措施，帮助计划生育农户增加经济收入，解决实际困难，提高社会经济地位。各级政府及扶贫开发部门应有计划、有重点地对实行计划生育的贫困户予以优先扶持，提高他们的生产自救和发展能力。农村继续实行“增人不增地，减人不减地”的政策。在现阶段，对违反计划生育政策的家庭征收社会抚养费，给予必要的经济制约，收费标准由各省、自治区、直辖市统一制定。征收的社会抚养费上缴国家财政。
　　各级政府及基层组织要建立激励机制，落实对实行计划生育家庭的奖励和优惠政策。对独生子女户发给一定数量的奖励费，城市独生子女父母退休时，各地可根据实际情况给予必要的补助。对实行计划生育的家庭特别是只有女孩的家庭，在分配集体经济收入、享受集体福利、划分宅基地、承包土地、培训、就业、就医、住房及子女入托、入学等方面给予适当照顾。
　　积极发展社会保障事业，解除群众实行计划生育的后顾之忧。劳动保障及其他有关部门要从实际出发，通过多种途径，采取优惠措施，建立有利于计划生育的社会保障制度。在城市，积极建立并发展养老保险、基本医疗保险、生育保险和社会福利等社会保障制度；在农村，坚持政府支持和农民自愿的原则，根据实际情况逐步建立实行计划生育的独生子女户和两女户的养老保障制度。保证生育妇女的劳动权利和经济利益。民政等部门要制定有利于计划生育家庭的社会救助政策，对实行计划生育的贫困户给予生活保障补助。
　　10．将人口问题纳入西部大开发战略的总体规划。实施西部大开发战略，必须坚持发展经济和控制人口两手抓，做到经济要上去，人口数量要下来，人的素质要提高。针对西部地区生育率较高、贫困人口比例较大、城市化水平较低的状况，制定相关政策和措施，加强计划生育基层基础工作，严格控制人口增长。国家采取财政转移支付制度和实施扶贫攻坚战略，大力发展教育、科技、文化、医疗卫生和计划生育事业。实施东部支持西部，城市支援农村，先进帮助后进的对口援助措施。加强干部培训，注重工作指导，改善服务设施，提高服务水平。

　　**四、**建立适应社会主义市场经济体制的人口与计划生育工作管理机制
　　11．人口与计划生育工作是一项政策性强、涉及广大群众切身利益的社会系统工程，各级党委和政府要协调有关部门，动员全社会力量实行综合治理。纪检监察、组织、宣传和计划、教育、科技、民族、公安、民政、司法、财税、人事、劳动保障、建设、国土资源、农业、文化、卫生、广播电视、统计、工商等部门要进一步明确在人口与计划生育工作中的职责和任务，制定具体措施。计划生育部门要充分发挥职能作用，协助党委和政府组织、协调、督促、推动各部门落实人口与计划生育政策。各级工会、共青团、妇联和计划生育协会等群众团体作为协助党委、政府动员广大群众参与计划生育工作的重要力量，要更好地组织群众实行自我教育、自我管理、自我服务，积极发挥作用。
　　12．积极创造条件，把人口与计划生育工作纳入社区基层管理和服务体系。在农村，把计划生育工作作为农村基层组织建设和“小康村”、“文明村”、“五好文明家庭”建设的重要内容，开展创建计划生育合格村活动，积极推进村民自治，实行计划生育村务公开、民主管理、民主监督。在城市，继续推行属地化管理，强化街道办事处、居委会计划生育的管理职责，积极推进社区建设，开展社区服务。
　　加强对流动人口的综合管理。按照《[流动人口计划生育工作管理办法](https://www.pkulaw.com/chl/c8062869d0fb3652bdfb.html)》的规定，在流入地和流出地共同负责的基础上，坚持以流入地管理为主，公安、工商、税务、劳动保障、卫生、房产管理等有关部门要切实履行职责，围绕办证、租房、用工等环节，在现居住地形成有效的管理和服务网络，努力为流动人口提供多方面的服务。
　　进一步落实法定代表人计划生育工作责任制。各类所有制形式的企业和事业单位、各种社会组织等都要依据有关法律法规，承担管理计划生育的责任，落实计划生育工作的人员、经费和措施以及计划生育的奖励优惠政策。
　　13．宣传、教育、科技、文化等部门要密切配合，利用各种传媒，采用多种方式，大力开展人口与计划生育基础知识的公益性宣传，在全社会形成有利于计划生育的良好氛围。深入开展“婚育新风进万家”和文化、科技、卫生“三下乡”活动，大力宣传和普及避孕节育、优生优育、生殖保健的科学知识，引导广大群众树立晚婚晚育、少生优生、生男生女都一样等科学、文化、进步的婚育观，努力建设社会主义新型生育文化。各级党校、行政干部学校、团校等要重视人口与计划生育教育工作，中等以上学校普遍开设人口及青春期、性保健讲座或课程。
　　14．开展以技术服务为重点的优质服务。计划生育部门是人口与计划生育技术服务综合管理部门，计划生育和卫生部门要根据各自职责，密切配合，围绕生育、节育、不育共同做好计划生育技术服务和生殖保健服务。基层计划生育技术服务人员要深入千家万户，指导育龄群众选择以长效避孕措施为主的安全、有效、适宜的避孕方法。各级政府要加强对县（市）、乡（镇）、村计划生育技术服务机构和医疗、保健机构服务网络的管理，充分利用现有卫生资源，改善服务条件，规范服务标准，增强服务能力。
　　15．依靠科技进步，发展计划生育事业。加快人口与计划生育领域的科学研究和技术创新工作。开发、推广避孕节育、优生优育、生殖保健的新技术和新产品，发展生殖健康产业。加强计划生育药具及保健用品市场管理，规范市场行为。
　　16．加强人口与计划生育领域的国际合作与交流。不断拓宽合作领域，积极参与国际人口与计划生育领域的多边及双边活动，学习和借鉴国际社会的有益经验和科学方法，加强对外宣传，树立我国人口与计划生育工作的良好形象，争取国际社会广泛的理解和支持。

　　**五、**切实加强党和政府对人口与计划生育工作的领导
　　17．党和政府的坚强领导是做好人口与计划生育工作的根本保证。各级党委和政府要把做好人口与计划生育工作摆到可持续发展战备的首要位置，坚持党政一把手亲自抓、负总责。认真研究新情况，协调制定符合本地区实际的工作计划，组织各方面力量抓好落实，切实做到责任到位，措施到位，投入到位。要充分发挥国家计划生育委员会兼职委员及其单位和各省、自治区、直辖市人口与计划生育领导小组的作用。中央每年召开一次人口工作座变会，把人口与计划生育工作纳入中央重大事项的督查范围，不定期地对各省、自治区、直辖市进行专题调查和重点检查。各省、自治区、直辖市党委和政府每年要将本地区人口与计划生育工作情况向中央作专题报告。
　　党政干部和计划生育干部要提高自身素质，增强法律意识和法制观念，切实转变工作作风，密切联系群众，关心群众生活，帮助群众解决实际困难，认真做好深入细致的思想工作；要带头执行国家计划生育政策，不得利用职权超生。干部超生的，一律给予政纪处分，不能担任领导职务；党员超生的，给予党纪处分。
　　18．坚持和完善人口与计划生育目标管理责任制，对党政领导和计划生育部门分别进行责任考核，落实“一票否决”制度。组织、纪检监察等部门要把领导干部落实计划生育责任制的情况作为衡量政绩和选拔奖惩的重要内容，任期内逐年考核，离任时进行审核，对工作失职的要追究责任。对人口与计划生育工作的考核要突出重点，注重实效，简便易行，把基层的注意力和工作重点引导到为群众提供优质服务、提高工作水平上来。
　　19．各级党委和政府及组织、人事部门要按照思想好、作风正、懂业务、会管理、善于做群众工作的要求，加强计划生育干部队伍特别是乡（街道）、村（居委会）基层计划生育干部队伍建设，落实人员、任务、报酬，从政治上、生活上关心爱护计划生育干部，为他们开展工作创造条件，确保计划生育工作机构的稳定。要加强对计划生育工作人员的教育、培训，提高他们的政治、业务素质和职业道德水准，进强法制观念、群众观念和服务观念。改革干部人事制度，选择优秀人才充实计划生育工作队伍。
　　20．人口与计划生育工作是一项具有显著社会效益的公益性事业，对人口与计划生育工作的投入属于国家经济社会发展的基础性投入。要把计划生育经费纳入各级政府的财政预算，切实予以保证。逐步提高人口与计划生育经费投入的总体水平，计划生育事业费增长幅度高于财政收入的增长幅度，到2005年末，各级财政投入计划生育事业费年人均超过10元。对中西部、少数民族及灾区等财政困难地区的计划生育事业费实行投入倾斜。农村税费改革过程中，应切实保障基层计划生育事业费的必要投入。社会抚养费、乡统筹费纳入财政预算后，财政相应增加计划生育经费的投入。建立多渠道的筹资体制，鼓励民间捐资、社会募捐和国际捐赠。国家支持建立人口与计划生育公益基金。审计部门要加强监督检查，严肃查处社会扶养费征收、管理和使用中出现的问题。
　　中国人民解放军和中国人民武装警察部队的计划生育工作，参照本决定精神，由中国人民解放军和中国人民武装警察部队另行制定。
　　各级党委和政府要结合本地区实际，创造性地做好人口与计划生育工作，在以江泽民同志为核心的党中央领导下，高举邓小平理论伟大旗帜，抓住机遇，迎接挑战，振奋精神，真抓实干，把我国的人口与计划生育事业全面推向新阶段，为实现社会主义现代化建设的宏传目标做出更大的贡献。

© 北大法宝：（[www.pkulaw.com](https://www.pkulaw.com/)) 专业提供法律信息、法学知识和法律软件领域各类解决方案。北大法宝为您提供丰富的参考资料，正式引用法规条文时请与标准文本核对。
欢迎查看所有[产品和服务](https://www.pkulaw.com/helps/2.html)。法宝快讯：[如何快速找到您需要的检索结果？](https://www.pkulaw.com/helps/70.html)[法宝](https://www.pkulaw.com/helps/69.html)V6有何新特色？

扫描二维码阅读原文

**原文链接：**<https://www.pkulaw.com/chl/f827fcaffffe8d79bdfb.html>
